# Red Tape in Nursing Homes

**Published:** 1923-06

**Authors:** 


### Red Tape in Nursing Homes.

SIR,—Your corespondent "R. T. H." inquires why hospital routine, often galling in the extreme, must be "slavishly imitated" by the authorities of nursing homes. I should be glad to know why another great fault of our hospitals is repeated in nursing homes. I refer to the underfeeding of the nursing staff. It is, I believe, a generally accepted fact that few, if any, nurses have sufficient to eat during their period of training; but until recently I imagined that the state of affairs in the average nursing home was better, if not entirely satisfactory.

Experience has undeceived me, and I know of two or three large and expensive institutions where the nurses have barely sufficient food to sustain them, and that food both ill-chosen and unappetising. Surely the fees demanded by high-class nursing homes are sufficient to permit of the nurses being adequately and wholesomely fed, especially if it be remembered that not only are the nursing staff responsible for the welfare and general health of the patients, but also that a large amount of really hard manual labour falls to their lot, in addition to the long hours on duty, which appear to be a matter of absolute necessity.

K. A. H.

---

### Cocaine Substitutes Committee.

The Home Office and the Ministry of Health have had under consideration the possibility of the use in medical and surgical practice of substances which might serve the same therapeutic purpose as cocaine, but be free from its deleterious properties. The Minister of Health has decided, after consultation with the Medical Research Council, to appoint a committee to investigate the comparative value, for the therapeutic purposes for which cocaine is at present used, of various possible substitutes, and the evidence as to risk, if any, of such substitutes becoming drugs of addiction. The chairman is Dr. J. Smith Whitaker, a senior medical officer of the Ministry, and Sir William Henry Willcox is the Home Office representative. The secretary is Dr. E. W. Adams, O.B.E., a medical officer of the Ministry.

